# Lifestyle patterns associated with common mental disorders in Brazilian adolescents: Results of the Study of Cardiovascular Risks in Adolescents (ERICA)

**DOI:** 10.1371/journal.pone.0261261

**Published:** 2021-12-14

**Authors:** Sara Araújo Silva, Ariene Silva do Carmo, Kênia Mara Baiocchi Carvalho

**Affiliations:** 1 Faculty of Health Sciences, Graduate Program in Human Nutrition, University of Brasilia, Brasilia, Brazil; 2 Department of Nutrition, Unieuro University Center, Brasilia, Brazil; 3 Ministry of Health, Brasilia, Brazil; Addis Ababa University, ETHIOPIA

## Abstract

The association between lifestyle factors and mental health has been evaluated in isolation; however, there has been a lack of information about lifestyle patterns and Common Mental Disorders (CMD) in adolescents. Therefore, the present study aims to assess the association between sets of lifestyle patterns and the occurrence of CMD in Brazilian adolescents evaluated in a national school-based cross-sectional survey. The outcome variable considered was presence of CMD. Lifestyle patterns were identified from the Principal Component Analysis. Consumption of foods, water and alcoholic beverages, sleep, physical activity, and smoking were used to identify patterns as explanatory variables. Sociodemographic characteristics, administrative dependence of the school and, nutritional status, were considered adjustment factors in the regression model. A total of 70,427 adolescents were evaluated. The principal component analysis identified three lifestyle patterns: high consumption of ultra-processed foods and low consumption of unprocessed or minimally processed foods (pattern 1); high consumption of alcoholic beverages and tobacco in the last 30 days (pattern 2); and high consumption of water and high level of physical activity (pattern 3). In the adjusted model, in patterns 1 and 2, the third tertile presented greater chance of CMD (OR 1.68; CI 95% 1.51–1.87 and OR 1.38; CI 95% 1.19–1.60, respectively). In pattern 3, the second (OR 0.88; CI 95% 0.80–0.96) and the third (OR 0.80; CI 95% 0.72–0.88) tertiles presented lower chances of CMD among the adolescents evaluated. Therefore, we suggest that health-promoting practices aimed at adolescents include multiple behaviors, with the objective of ensuring physical, mental, and social well-being.

## Introduction

Teenage years require attention due to the profound changes in growth and development, influenced by environmental, nutritional and social factors that can impact the adolescents physical and mental well-being [1]. Psychological distress in adolescents has been shown to be a part of the scope of chronic non-communicable diseases that are progressively increasing worldwide [2]. In addition, around 70% of deaths related to preventable diseases, including chronic non-communicable diseases [3], are linked to risk factors that start at this stage of life [1].

The presence of Common Mental Disorders (CMD) leads to psychological distress in various dimensions of day-to-day life, such as the inability to manage thoughts, emotions, behaviors and social relationships [4]. CMDs are mostly characterized by the occurrence of anxiety and depression disorders [5], although it includes several other categories of commonly reported mental disease that impact individual mood and feelings [6].

The diagnosis of mental disorders can occur through standardized diagnostic interviews, as recommended by the American Psychiatric Association [6], or with the use of rapid screening scales [7]. However, the identification of CMD does not imply a formal psychiatric diagnosis. The General Health Questionnaire (GHQ), in its 12-item version, is a fast tracker used in research to measure psychological well-being [6] or psychological distress, such as anxiety and depression [7]. A systematic review of observational studies from different countries found a prevalence of CMD in adolescents of 31.0% (CI 95% 28.0–34.0; I^2^ = 97.5%), based on a GHQ-12 cut-off point of 3 or more symptoms [8]. Using the same cut-off point, a very similar result was observed in Brazil through the application of the Study of Cardiovascular Risks in Adolescents (ERICA), which found a prevalence of CMD of 30.0% (CI 95% 29.2–30.8) [9].

Through a systematic review, longitudinal studies indicated an association between lifestyle and depression in adolescents [9]. The effects of risk and protective factors on CMD, including diet quality, alcohol consumption, smoking, sleep duration and physical inactivity, have been studied by several authors. Molendijk et al. [10], for example, demonstrated that a better diet quality was associated with a lower risk of developing depressive symptoms, and Sarris et al. [11], concluded that nutrition is a modifiable factor, both in preventing mental disorders and in promoting mental health.

In a study based on the National School Health Survey, carried out in 2015 in Brazil, a higher risk of anxiety-induced sleep disorder was observed among adolescents who simultaneously presented high intake of ultra-processed foods and high sedentary behavior. Negative lifestyles are associated with a substantial increase in the risk of anxiety-induced sleep disorders [12]. Although previous studies have shown associations between one or a few lifestyle factors and mental health, adolescent population-based studies using an approach constructed on patterns that consider simultaneously a set of risk factors (food and alcoholic consumption, sleep patterns, physical activity, and smoking) in association to mental health are scarce. Therefore, to better understand the factors associated with CMD, the present study aims to assess the connection between sets of lifestyle patterns and CMD in Brazilian adolescents.

## Methods

We followed the guidelines of the document “Recommendations of the Strengthening the Reporting of Observational Studies in Epidemiology (STROBE)” in the writing of this manuscript [13].

### Study design

This research analyzed data from the Study of Cardiovascular Risks in Adolescents (ERICA), a cross-sectional, national, school-based study, conducted from February 2013 to November 2014, with the objective of estimating the prevalence of cardiovascular risk factors in adolescents aged from 12 to 17 years, who attended schools in Brazilian municipalities with more than 100 thousand inhabitants [14].

### Sample size

The ERICA sample was gathered from schools, school periods (morning or afternoon), grades, and classes, and was calculated to estimate 12 domains with controlled precision, considering sex and age. Initially, the study established 32 geographic strata (26 capitals, the Federal District and five sets composed of other municipalities in each macro-region of the country) according to the characteristics of the 2009 School Census. The calculation of the complex sample was carried out in three levels. On the first level, the selection of the school sample was carried out based on probability proportional to size. The sample size corresponded to the ratio between the number of students in the considered school periods and grades, and the distance, measured in kilometers, between the seat of the municipality where the school was located and the seat of the capital city. Using this measure, 1,251 schools located in 124 municipalities were selected by the ERICA. On the second level of selection, three classes were identified in each school considering the period (morning or afternoon) and grade (7th, 8th and 9th grade of elementary school or 1st, 2nd, and 3rd grade of high school). This level of selection was based on the correspondence between the adolescents’ grades and the targeted age for the study. Finally, on the third level of selection, all selected students from the three classes of each school were invited to participate in the study. Therefore, the sample was probabilistic and representative of the country, the five regions, and the state capitals. More information on the ERICA sampling methodology are detailed by Vasconcellos et al. [15].

From 102,327 eligible adolescents, 23.7% (24,284) did not answer any of the data collection blocks. The self-administered questionnaire was answered by 74,589 students and 73,160 answered the 24-hour dietary recall. Students that were outside the age group, pregnant or that presented physical or mental disability (considered to be any temporary or permanent disability that prevented the carrying out of research measures) were considered ineligible for the study [16].

**Fig 1:**
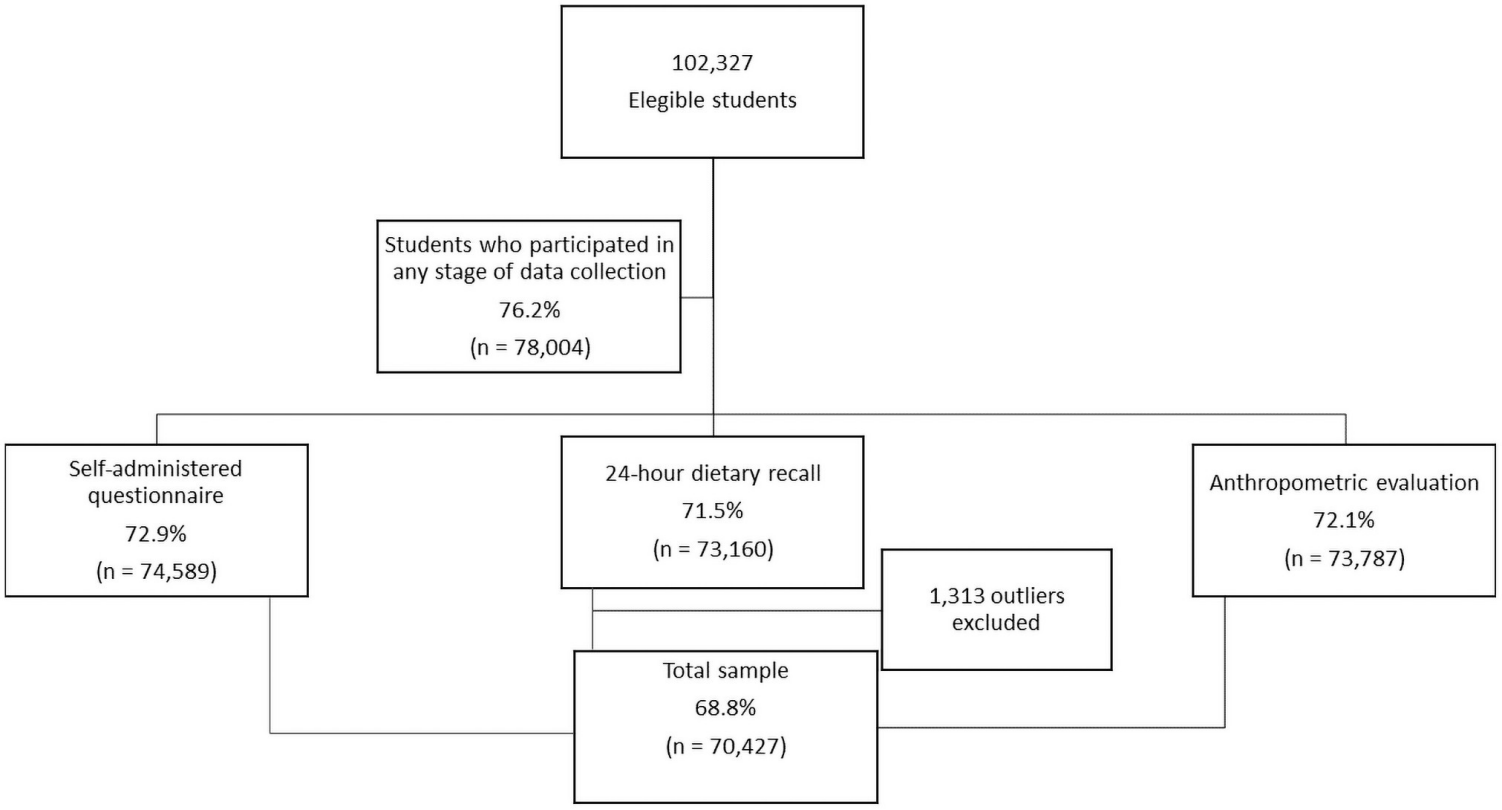
Flowchart of eligible adolescents and the total study sample.

In this study, the sample was composed of adolescents who fully answered the mental health assessment available in the questionnaire and who presented the data from the first 24-hour dietary recall and anthropometric data. A total of 1,313 adolescents were further excluded because they reported intakes under 500 kcal or above 6,000 kcal [17]. The final sample of 70,427 adolescents corresponded to a response rate of 68.8% (Fig 1).

### Data collection

The self-administered questionnaire was made available on the Personal Digital Assistant (PDA), containing questions about sociodemographic characteristics, alcohol consumption, sleep patterns, physical activity, smoking, as well as dietary practices and a mental health assessment.

The 24-hour dietary recall was carried out by interviewers who recorded all the information about the food and beverages consumed in an exclusive software for ERICA [18] using the Multiple Pass Method [19]. This software, called ERICA-REC24, included a list of foods and beverages according to the Brazilian Food Composition Table (in portuguese: *Tabela de Composição Nutricional dos Alimentos Consumidos no Brasil*) [14].

Body weight was measured using a digital scale made by the brand Líder, model P150 (São Paulo, Brazil), with an accuracy of 50 g and a maximum capacity of 200 kg. Height was assessed using a portable stadiometer made by the brand Alturexata (Minas Gerais, Brazil), with an accuracy of 1 mm and a maximum capacity of 213 cm.

To minimize the risk of bias, there was no interference during the period in which the adolescents were answering the questionnaire on the PDA, and previously trained researchers performed the collection of the food consumption data and the anthropometric data.

### Variables

#### Outcome variable

The outcome variable was presence of CMD, measured by the 12-item version of the General Health Questionnaire (GHQ-12) [20,21]. The responses were organized on a scale with four options. For questions that presented positive mental health aspects, the possible alternatives were: (1) more than usual, (2) the same as usual, (3) less than usual, and (4) much less than usual; for negative aspects, they were: (1) nothing, (2) no more than normal, (3) a little more than normal, and (4) much more than normal.

The positive aspects were identified by the following questions: “Have you been able to maintain your attention on the things you are doing? Have you felt useful in most of your daily activities? Have you been able to face your problems? Have you been able to make decisions? Have you been feeling happy in general? Have you been satisfied with your daily activities?”

The negative aspects were observed from the following questions: “Have you been losing a lot of sleep due to worry? Have you been feeling constantly nervous and tense? Have you felt that it is hard to overcome your difficulties? Have you been feeling sad and depressed? Have you lost confidence in yourself? Have you been considering yourself a worthless person?”

The proposed questionnaire score was determined by the standard system (0-0-1-1), allowing a variation from zero to 12. The higher the total score, the more severe the state of mental health. In this sense, we considered the presence of CMD when a score was equal to or greater than three [22].

#### Independent variables

Based on the first 24-hour dietary record, we carried out food conversions, in grams, and all 1,015 food items were identified according to the NOVA classification and organized to obtain the adolescents’ individual daily consumption. The NOVA classification groups foods according to the extent and purpose of industrial processing and establishes four food groups: unprocessed or minimally processed foods; culinary ingredients; processed foods; and ultra-processed foods [23].

Based on NOVA’s identification of the 4 food groups, two variables were established: consumption of ultra-processed foods and consumption of unprocessed or minimally processed foods, both expressed through continuous variables as grams by total energy intake (kcal) per day. Energy intake was estimated based on the Brazilian Table of Food Composition [14,24] and expressed in kcal. Then, the amount of food consumed in grams was calculated according to the total energy intake of the diet and transformed into consumption quartiles. The consumption quartiles of ultra-processed foods and unprocessed or minimally processed foods were expressed in grams by total energy intake per day.

The variable water consumption was obtained from the question “How many glasses of water do you drink a day?” and categorized into do not drink water, 1 to 2 glasses a day, 3 to 4 glasses a day, and 5 or more glasses a day.

The consumption of alcoholic beverages was obtained from the question “In the last 30 days (one month), on how many days did you have at least one glass or one dose of an alcoholic beverage?” The categories of analysis were: “never had an alcoholic beverage”, “did not drink in the last 30 days”, “less than one glass or dose”, “1 glass or dose”, “2 glasses or doses”, “3 glasses or doses”, “4 glasses or doses” or “5 or more glasses or doses in the last 30 days”.

The adequate sleep variable was obtained from the weighted average of sleep duration during the weekdays and on weekends, and was classified into two categories: no, when the daily sleep hours were less than 8 or greater than or equal to 11 hours of sleep, or yes, when ranging from 8 to 10 hours of sleep [25].

The variable physically active was obtained through the application of the Physical Activity Questionnaire for Adolescents (QAFA) [26]. Three categories of analysis were considered according to the indicated time of activity in the seven days prior to the survey, the categories were: inactive (0 minutes/week), insufficiently active (1–299 minutes/week) or active (300 or more minutes/week) [27,28].

Smoking was assessed based on the question “In the last 30 days, on the days you smoked, how many cigarettes did you have?”, and the answers were categorized as “never smoked”, “have not smoked in the last 30 days”, “less than 1 cigarette a day”, “1 cigarette a day”, “2 to 5 cigarettes a day”, “6 to 10 cigarettes a day” or “more than 11 cigarettes a day”.

#### Adjustment variables

The adjustment variables included in the regression model were sociodemographic characteristics and nutritional status.

The sociodemographic characteristics analyzed were as follows: sex (female or male), age range (12 to 14 years or 15 to 17 years), race/color (white, black or brown, and Asian or indigenous) and administrative dependence of the school (public or private).

The classification of nutritional status was obtained from the body mass index for age and sex, according to the World Health Organization [29]. The following cut-off points were used: underweight (z-scores < -2), adequate (z-scores ≥ -2 and ≤1), overweight (z-scores > 1 and ≤ 2) and obesity (z-scores > 2).

### Statistical analysis

Regarding the data analysis, we performed a descriptive analysis using the calculation of the distributions of relative frequencies and averages for categorical and quantitative variables, respectively, with their corresponding 95% confidence intervals. Chi-square test and Student t-test were used to compare proportions and averages, respectively.

Variables related to lifestyle, such as consumption of ultra-processed foods, consumption of unprocessed and minimally processed foods, water consumption, consumption of alcoholic beverages, sleep, physical activity, and smoking served as the basis for the identification of the patterns used as explanatory variables, while sex, age, race/color, administrative dependence of the school and nutritional status, were considered adjustment factors in the regression model.

We obtained the lifestyle patterns from the Principal Component Analysis (PCA), which corresponds to a multivariate technique used in the identification of patterns, components, or factors. This technique allows the reduction of the number of variables to maximize the power of explanation for the data set.

We applied the varimax orthogonal rotation method and the Kaiser-Meyer-Olkin index (KMO) was used to assess the factorability of the data, adopting values between 0.5 and 1.0 as acceptable for this index [30]. In the analysis of standardized estimates, we considered factor loads greater than |0.3| and p < 0.05 for the construction of latent variables, as an indication that the correlation between the observed variable and the constructor was moderately high in magnitude [31,32].

The number of patterns to be extracted was defined by eigenvalues > 1.0 and lifestyle patterns were generated in continuous variables. The variables of the identified patterns were categorized according to the distribution of tertiles.

The effect of lifestyle patterns on CMD was observed, initially, through a bivariate logistic regression analysis. Then, we performed a multiple logistic regression analysis, considering the adjustment for the variables (sociodemographic characteristics and nutritional status). Odds Ratio (OR) with a 95% confidence interval was used as a measure of effect and p-values <0.05 were considered statistically significant.

We processed statistical analyzes using version 14.0 of the Stata software (StataCorp LP, College Station, United States), which possesses a module called Survey for analyzing complex sample data. This module considered the complexity of the ERICA sample and presented specific commands for adjusting models and calculating dispersion measures.

### Ethical aspects

ERICA was conducted according to the guidelines determined by the Declaration of Helsinki and all procedures involving the research study participants were approved by the Human Research Ethics Committee of the Federal University of Rio de Janeiro in 2009 (protocol number 45/2008). The adolescents submitted a written term signed by themselves, as recommended by the Ethics Committee.

## Results

The characteristics of the sample are summarized in Table 1. Of a total of 70,427 adolescents evaluated, 50.2% were male, 52.7% aged between 12 and 14 years, 57.2% were black or brown, and 82.6% studied in public schools. Regarding nutritional status, 0.4% were underweight, 17.1% were overweight and 8.4% were obese.

**Table 1:** Distribution of adolescents in relation to common mental disorders, sociodemographic characteristics, nutritional status, and lifestyle characteristics. Study of Cardiovascular Risks in Adolescents (ERICA), Brazil, 2013–2014.

Variables	All		Common mental disorders prevalence		
	%	CI 95%	%	CI 95%	p-value
**Sociodemographic characteristics**					
**Sex**					<0.001
Female	49.8	*	38.3	37.0–39.6	
Male	50.2	*	20.9	19.7–22.2	
**Age range**					<0.001
12 to 14	52.7	*	26.3	25.0–27.5	
15 to 17	47.3	*	33.2	31.9–34.6	
**Race/color** ^†^					<0.05
White	40.1	38.4–41.7	29.2	27.6–30.8	
Black or brown	57.2	55.5–58.8	29.4	28.4–30.4	
Asian or indigenous	2.8	2.5–3.1	34.9	31.4–38.4	
**Administrative dependence of the school**					0.621
Public	82.6	78.3–86.8	29.5	28.4–30.6	
Private	17.4	13.2–21.7	29.9	28.7–31.0	
**Nutritional status** ^‡^					<0.05
Underweight	0.4	0.3–0.6	13.8	8.0–19.7	
Adequate	74	73.0–75.1	29.5	28.3–30.6	
Overweight	17.1	16.3–17.9	30.7	29.2–32.3	
Obesity	8.4	7.9–8.9	28.9	26.2–31.6	
**Lifestyle characteristics**					
**Consumption of ultra-processed foods (grams/kcal)**					<0.05
Quartile 1	24.2	22.8–25.6	27.9	26.0–29.8	
Quartile 2	23.3	22.6–24.1	28.0	26.2–29.8	
Quartile 3	25.2	24.3–26.1	30.5	29.0–32.1	
Quartile 4	27.3	26.2–28.5	31.5	30.2–32.8	
**Consumption of unprocessed or minimally processed foods (grams/kcal)**					<0.001
Quartile 1	24.4	23.1–25.6	33.0	31.4–34.5	
Quartile 2	24.2	23.5–25.0	29.8	28.2–31.3	
Quartile 3	24.4	23.7–25.1	28.0	26.7–29.4	
Quartile 4	27.0	25.4–28.6	27.7	25.6–29.8	
**Water consumption**					<0.001
Does not drink water	1.5	1.3–1.7	55.7	50.1–61.3	
1 to 2 glasses a day	18.7	17.8–19.5	36.7	34.6–38.8	
3 to 4 glasses a day	31.6	30.6–32.5	29.2	27.6–30.9	
5 or more glasses a day	48.2	47.1–49.4	26.2	25.0–27.4	
**Consumption of alcoholic beverages**					<0.001
Never had an alcoholic beverage	52.6	51.7–53.5	23.8	22.8–24.9	
Did not drink in the last 30 days	25.2	24.3–26.1	31.9	30.2–33.7	
Less than 1 glass or dose	4.6	4.2–5.0	34.7	29.8–39.7	
1 glass or dose	5.5	4.8–6.1	39.1	34.8–43.4	
2 glasses or doses	3.6	3.3–3.9	42.8	38.2–47.5	
3 glasses or doses	2.5	2.2–2.8	46.2	41.0–51,5	
4 glasses or doses	2.1	1.9–2.4	40.3	35.0–45.6	
5 or more glasses or doses	3.9	3.5–4.2	43.4	39.5–47.2	
**Adequate sleep** ^§^					<0.001
No	54.3	52.9–55.6	32.4	31.3–33.5	
Yes	45.7	44.4–47.1	26.2	24.9–27.5	
**Physically active** ^**||**^					<0.001
Inactive	26.6	25.8–27.4	36.4	35.2–37.6	
Insufficiently active	27.7	26.7–28.6	28.1	26.2–30.0	
Active	45.7	44.7–46.7	26.5	25.1–27.8	
**Smoking**					<0.001
Never smoked	84.4	83.6–85.2	29.5	28.6–30.3	
Did not smoke in the last 30 days	11.2	10.5–11.8	48.4	42.8–54.0	
Less than 1 cigarette a day	1.4	1.2–1.6	27.0	26.1–28.0	
1 cigarette a day	1.2	1.0–1.4	42.1	40.2–43.9	
2 to 5 cigarettes a day	1.2	1.0–1.3	44.1	37.0–51,2	
6 to 10 cigarettes a day	0.4	0.3–0.5	41.3	32.8–49.7	
More than 11 cigarettes a day	0.3	0.2–0.4	42.8	30.0–59.7	

CI, Confidence interval.

*Variables used to calculate the natural weights and calibration factors of the sample.

^†^Not reported by 1,867 adolescents.

^‡^ Underweight, z-scores <-2; adequate, z-scores ≥-2 and ≤1; overweight, z-scores >1 and ≤2; obesity, z-scores >2).

^**§**^No, <8 hours or ≥11 hours/day; Yes, ≥ 8 and < 11 hours/day.

^¶^ Inactive (0 minutes/week); insufficiently active (1–299 minutes/week); active (300 or more minutes/week).

Regarding the lifestyle characteristics, we found that the prevalence of CMD in quartiles 1 and 4 of consumption of ultra-processed food is 27.9% and 31.5%, respectively. And the prevalence of CMD in quartiles 1 and 4 of consumption of unprocessed or minimally processed foods is 33.0% and 27.7%, respectively. The consumption of 5 or more glasses of water was reported by 48.2% (CI 95% 51.7–53.5), 52.6% never had an alcoholic beverage and 25.2% had not had an alcoholic beverage in the last 30 days. 45.7% had adequate sleep (8 to 10 hours) (CI 95% 44.4–47.1), 45.7% had sufficient physical activity (CI 95% 44.7–46.7) and 84.4% were not smokers (CI 95% 83.6–85.2).

The analysis of the PCA allowed the identification of three lifestyle patterns that could potentially present interesting associations. Table 2 shows the factor loading of the patterns 1, 2 and 3. In the first test to identify the patterns, the variable adequate sleep was included in the PCA and presented a factor loading <|0.3|, leading to a 56.1% of explained variance. When removed from the model, we observed an increase in the percentage of explained variance, which contributed to 64.8% of the variance of the total information.

**Table 2:** Factor loading of principal component analysis (PCA) in Brazilian adolescents. Study of Cardiovascular Risks in Adolescents (ERICA), Brazil, 2013–2014.

Lifestyle characteristics	Pattern 1	Pattern 2	Pattern 3	KMO
Consumption of ultra-processed foods	**0.576** *	-0.306	0.266	0.509
Consumption of unprocessed or minimally processed foods	**-0.548** *	0.321	-0.317	0.507
Water consumption	-0.259	0.199	**0.626** *	0.516
Physically active	-0.125	0.294	**0.642** *	0.505
Consumption of alcoholic beverages	0.386	**0.579** *	-0.108	0.507
Smoking	0.370	**0.585** *	-0.115	0.507
Eigenvalue	1.5	1.3	1.1	-
Explained variance (%)	24.3	22.0	18.6	-
Cumulative variance explained (%)	24.3	46.3	64.8	-
Overall	-	-	-	0.508

KMO, Kaiser-Meyer-Olkin.

*Factor loading > |0.3| made up the lifestyle patterns for the logistic regression analysis.

The KMO index and the factor loadings of all indicators were satisfactory (factor loading > |0.3|). Thus, pattern 1 was characterized by a high consumption of ultra-processed foods (factor loading = 0.576) and low consumption of unprocessed or minimally processed foods (factor loading = -0.548). Pattern 2 was characterized by high consumption of alcoholic beverages (factor loading = 0.579) and an increase in the number of cigarettes per day in the last 30 days (factor loading = 0.585). And pattern 3 was composed of high consumption of water (factor loading = 0.626) and high level of physical activity (factor loading = 0.642).

Table 3 shows the simple and multiple logistic regressions for CMD according to lifestyle patterns in Brazilian adolescents. In the simple logistic regression, pattern 1 revealed that in the second tertile there were less chances of CMD (OR 0.92; CI 95% 0.86–0.99) when compared to the reference category. However, the odds of CMD were higher in the third tertile (OR 1.67; CI 95% 1.53–1.82). In pattern 2, we observed the same as in pattern 1, but the third tertile had a greater chance of CMD (OR 1.37; CI 95% 1.24–1.52). Regarding pattern 3, in the third tertile (OR 0.69; CI 95% 0.64–0.76) there were less odds of CMD. In the adjusted model, in pattern 1 and 2, the third tertile presented greater chance of CMD. In the first pattern, the odds ratio was equal to 1.68 (CI 95% 1.51–1.87) and in the second pattern it was equal to 1.38 (CI 95% 1.19–1.60). Finally, in pattern 3, the second (OR 0.88; CI 95% 0.80–0.96) and the third (OR 0.80; CI 95% 0.72–0.88) tertiles presented lower odds of CMD among the adolescents evaluated.

**Table 3:** Crude and adjusted logistic regression models (95% CIs) for common mental disorders in Brazilian adolescents. Study of Cardiovascular Risks in Adolescents (ERICA), Brazil, 2013–2014.

Variables	OR* (CI 95%)	OR^†^ (CI 95%)
**Pattern 1**		
Tertile 1	Ref	Ref
Tertile 2	0.92 (0.86–0.99)*	1.22 (1.11–1.33) ***
Tertile 3	1.67 (1.53–1.82) ***	1.68 (1.51–1.87) ***
**Pattern 2**		
Tertile 1	Ref	Ref
Tertile 2	0.72 (0.67–0.77) ***	0.88 (0.78–0.99) *
Tertile 3	1.37 (1.24–1.52) ***	1.38 (1.19–1.60) ***
**Pattern 3**		
Tertile 1	Ref	Ref
Tertile 2	0.94 (0.86–1.02)	0.88 (0.80–0.96) **
Tertile 3	0.69 (0.64–0.76) ***	0.80 (0.72–0.88) ***

OR, Odds Ratio; CI, Confidence Interval; Ref, reference category.

*Simple ordered logistic regression model.

^†^OR adjusted for sex, age range, race/color, administrative dependence of the school and nutritional status.

^‡^ Pattern 1 characterized by high consumption of ultra-processed foods and low consumption of unprocessed or minimally processed foods.

^**§**^ Pattern 2 characterized by high consumption of alcoholic beverages and smoking.

^¶^ Pattern 3 characterized by high consumption of water and physical activity.

*p<0.05.

**p<0.01.

***p<0.001.

## Discussion

The present study allowed a broad and joint approach to the lifestyle components potentially associated with mental health, based on a robust sample of Brazilian adolescents. Three lifestyle patterns were identified: two patterns based on unhealthy characteristics (pattern 1 and 2), and another based on healthy characteristics (pattern 3). After adjusting for possible confounding variables, we observed that all patterns affected the chances of CMD. These results reinforced the importance of considering lifestyle characteristics not only for physical, but also mental health during the adolescence.

Although our study, like others, showed that the prevalence of CMD was higher among adolescents that presented inadequate sleep [33,34], this variable was not eligible to compose the standards, most likely because our study’s data collection protocol did not show sleep characteristics in detail. Therefore, we removed it from the analysis model.

The diet of the Brazilian population is traditionally characterized by the combination of rice and beans, but in the last years an increase in the consumption of ultra-processed foods has been observed, regardless of the population’s age group [35]. Of the total calories consumed by Brazilian adolescents, 28% come from ultra-processed foods [36]. The Food Guide for the Brazilian Population recommends that unprocessed or minimally processed foods be the basis of the diet, and that ultra-processed foods should be avoided in order to promote health [37].

The literature points out that higher consumption of ultra-processed foods favors the development of obesity and other chronic non-communicable diseases, increasing specially the risk of depressive symptoms [38–41]. Therefore, the presence of variables related to diet quality in lifestyle patterns presented here confirm the importance of the NOVA classification as an instrument of nutritional assessment. However, the association between food and mental health can be bidirectional, especially in terms of depression: people diagnosed with depressions can have a poor diet or a poor diet can favor the occurrence of depression [42].

The association between consumption of alcohol and tobacco, measured by the intake of at least one dose of alcoholic beverage and cigarettes, respectively, in the last 30 days, and the presence of CMD in the Brazilian adolescent population had already been reported [43], as well as the association between alcohol use and drunkenness, and the psychological distress of adolescents in different countries [44]. This fact is even more worrying, considering the high prevalence of alcohol consumption among adolescents in Brazil (approximately 24%) [45] and in different countries, such as the United States, which in 2019 found that the current alcohol use was of 29.2% among high school students [46].

In addition, a cross-sectional study of the Behavioral Risk Factor Surveillance System identified a strong association between the use of e-cigarettes and depression in the adult population of the United States. It was found that former and current cigarette users were more likely to report a history of clinically diagnosed depression when compared to those who never used cigarettes [47].

Pattern 3, which was composed of variables physical activity and consumption of water, revealed synergy between lifestyle related factors, since healthy and unhealthy characteristics were grouped in the same pattern. In this study, the prevalence of CMD was lower among physically active adolescents, a finding that corroborates with the evidence that physical activity has a protective effect against depressive symptoms, regardless of intensity. This finding also shows the social benefits of exercises and of the physical activity environments, which also favor the prevention of CMD [48,49].

The advantage of assessing behaviors as lifestyle patterns is to allow a broader approach to the problem and the synergy of different behaviors [32]. The adjustments applied to the statistical model for sociodemographic variables and nutritional status, consolidate the magnitude of the association between the investigated patterns and the outcome variable (CMD), and point to the urgency of a health promoting policy with a more comprehensive potential.

We believe that the combination of a response rate of approximately 70% for a questionnaire that addresses everyday perceptions and attitudes and that is used for CMD tracking, in a sample of more than 70,000 subjects, allows the identification of valid data such as those presented in this study.

However, some limitations must be pointed out. First, there was no way to guarantee that the adolescents fully understood the questions presented in the self-applicable questionnaire. Nevertheless, this measure ensured that there was no interference from the interviewer and was in accordance with the recommendation for the GHQ-12 application [21].

It is important to note that GHQ-12 is a screening tool and does not provide an accurate diagnosis of CMD, consequently, it may overestimate the prevalence of risk of stress and depression among adolescents. In addition, the age group for its precise validation was established for adolescents over 15 years of age and the presence of 4 symptoms was set as the cut-off point to CMD, while the ERICA data set considered adolescents from 12 years of age and the cut-off point of 3 symptoms to define presence of CMD.

The GHQ-12 is a self-applicable instrument and the various existing versions have been used as screening tools to identify the presence of CMD. Although the GHQ was primarily aimed at evaluating adults in primary and outpatient care [7], the GHQ-12 has been widely used to assess adolescents, as revealed in a manuscript entitled Common mental disorders prevalence in adolescents: A systematic review and metanalyses, which identified 43 studies that adopted the GHQ in adolescents aged 12 to 19 years in 19 countries [8] and, similarly to the other studies identified in that systematic review, ERICA used the version of the GHQ-12 that was translated for the Portuguese language and evaluated in a validation study, with a psychiatric interview structured as the gold standard and criteria of three or more symptoms to identify the presence of CMD, whose results showed sensitivity of 85.0% and specificity of 79.0% and area under the ROC (Receiver Operating Characteristics) curve of 0.87 [22].

Second, the 24-hour dietary recall has limitations inherent to the method, related to memory bias and the use of only one 24-hour dietary recall. Despite the large sample, it does not allow analysis of usual consumption in the same way that it has been analyzed in other studies [32,50]. However, the Multiple Pass Method was applied to reduce the underreporting of food consumption. The last limitation was the design of the cross-sectional study, which did not allow conclusions of cause and effect.

Despite the limitations, ERICA was a school-based study with high methodological rigor in field research, with questionnaire conferences, standardization in anthropometric measures, quality equipment and a previously trained team for data collection. Finally, the robust statistical analysis that considered the sample design of the study at all levels can also be considered a strong point and increase confidence in the results we presented.

## Conclusion

The pattern composed by unhealthy lifestyle characteristics indicated an increased chance of CMD while the pattern composed by high consumption of water and sufficient physical activity indicated lower chances of CMD.

We suggest that, in addition to identifying isolated risk or protection factors, it is paramount to assess the synergy between them and, most importantly, favor the adoption of healthy lifestyle habits during adolescence. We recommend that health-promoting practices aimed at adolescents include multiple behaviors, with the objective of ensuring physical, mental, and social well-being.

## Supporting information

S1 File (XLSX)
